# Is there enough evidence to stop using available and accessible antipsychotics such as haloperidol and promote the use of newer and more expensive drugs? What is the hope for populations that cannot afford them

**DOI:** 10.1192/j.eurpsy.2025.2067

**Published:** 2025-08-26

**Authors:** C. Carreño Glaria, G. Keane, K. Ibragimov, A. LLosa

**Affiliations:** 1 Médecins Sans Frontières, Barcelona, Spain; 2 Médecins Sans Frontières; 3 Epicentre, Paris, France

## Abstract

**Introduction:**

Doctors Without Borders works in humanitarian settings. In these settings, we have observed a notable movement away from first generation medications such as haloperidol towards second-generation antipsychotics, where these medications are available. We began to question whether the evidence clearly justified this and decided to contribute to the evidence.

**Objectives:**

To assess the clinical benefits and harms of haloperidol compared to olanzapine for people with schizophrenia and schizophreniaspectrum disorders.

**Methods:**

Searched the Cochrane Schizophrenia study-based register of trials, screened the references of all included studies. We contacted relevant authors of trials for additional information where clarification was required or where data were incomplete. The register was last searched on 14 January 2023.

**Results:**

We didn’t find a statistically significant difference between haloperidol and olanzapine in global state (RR 0.84, 95% CI 0.69 to 1.02), nor in relapse (RR 1.42, 95% CI 1.00 to 2.02). Haloperidol resulted in an increase of extrapyramidal side effects compared to olanzapine (RR 3.38, 95% CI 2.28 to 5.02). For weight gain, there may be a large reduction in the risk with haloperidol compared to olanzapine (RR 0.47, 95% CI 0.35 to 0.61). Haloperidol may result in an increase of leaving the study early compared to olanzapine (RR 1.99, 95% CI 1.60 to 2.47).

**Conclusions:**

Overall, the certainty of the evidence was low to very low for the main outcomes in this review, making it difficult to draw reliable conclusions. There is no clear difference between haloperidol and olanzapine in terms of global state and relapse. Different side effect profiles were noted. These findings should contribute to continue using haloperidol and olanzapine.

Many studies did not use equivalent doses of the two medications when they were compared. Most studies used comparatively higher doses of haloperidol compared to olanzapine.

**Disclosure of Interest:**

None Declared

